# Seasonal distribution of *Cryptosporidium* spp., *Giardia duodenalis* and *Enterocytozoon bieneusi* in Tibetan sheep in Qinghai, China

**DOI:** 10.1186/s13071-022-05442-0

**Published:** 2022-10-27

**Authors:** Fan Yang, Li Ma, Jing-min Gou, Hui-zhong Yao, Mei Ren, Bing-ke Yang, Qing Lin

**Affiliations:** 1 College of Veterinary Medicine, Northwest A&F University, Yangling, Shaanxi Province 712100 People’s Republic of China; 2 State Key Laboratory of Veterinary Etiological Biology, Lanzhou Veterinary Research Institute, Chinese Academy of Agricultural Sciences, Lanzhou, Gansu Province 730046 People’s Republic of China

**Keywords:** *Cryptosporidium* spp, *Giardia duodenalis*, *Enterocytozoon bieneusi*, Tibetan sheep, China

## Abstract

**Background:**

*Cryptosporidium* spp., *Giardia duodenalis* and *Enterocytozoon bieneusi* can cause important intestinal diseases in ruminants. However, data on the distribution of these three protozoan pathogens in Tibetan sheep are limited.

**Methods:**

We collected 761 fecal samples from Tibetan sheep across four seasons in Qinghai Province, China, and screened the samples for *Cryptosporidium* spp., *G. duodenalis* and *E. bieneusi* using PCR-based sequence analysis of the genes encoding 18S ribosomal RNA, triosephosphate isomerase and the internal transcribed spacer, respectively.

**Results:**

The positivity rates of *Cryptosporidium* spp., *G. duodenalis* and *E. bieneusi* in Tibetan sheep were 3.68% (28/761 samples), 1.58% (12/761) and 6.44% (49/761), respectively. Four species of *Cryptosporidium* were identified: *C. xiaoi* (*n* = 13 samples), *C. ubiquitum* (*n* = 8), *C. bovis* (*n* = 6) and *C. ryanae* (*n* = 1). Two *G. duodenalis* assemblages, namely the A (*n* = 2 samples) and E (*n* = 10) assemblages, were detected. Five zoonotic *E. bieneusi* genotypes were found: BEB6 (*n* = 21 samples), COS-I (*n* = 14), CHS3 (*n* = 11) and CGS1 (*n* = 2) from group 2, and PIGEBITS5 (*n* = 1) from group 1. Geographic differences in the distribution of *E. bieneusi*, and seasonal differences for all the three protozoan pathogens were noted.

**Conclusions:**

Our results elucidate the prevalence and genetic diversity of these three pathogens in Tibetan sheep across different regions and seasons, including zoonotic pathogens such as *C. ubiquitum*, *C. ryanae*, *G. duodenalis* assemblage A and five genotypes of *E. bieneusi*.

**Graphical Abstract:**

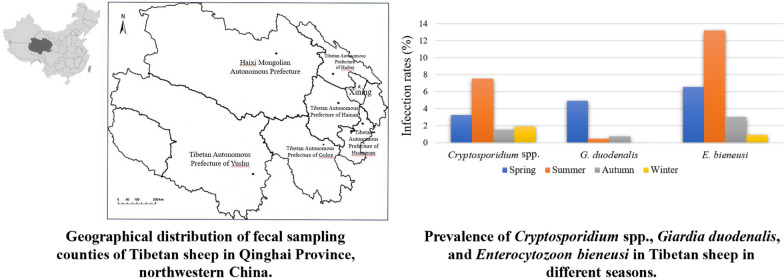

**Supplementary Information:**

The online version contains supplementary material available at 10.1186/s13071-022-05442-0.

## Background

*Cryptosporidium* spp., *Giardia duodenalis* and *Enterocytozoon bieneusi* are three important intestinal protozoa that can cause gastrointestinal discomfort and diarrhea in various hosts [1, 2]. The infections cause by these pathogens are self-limiting in healthy individuals, but in immunocompromised individuals, the infection period can be protracted, and even turn out to be life-threatening [3, 4]. To date, at least 42 *Cryptosporidium* spp. and 60 genotypes have been identified [5], with most of these species and genotypes being host-specific. *Giardia duodenalis* is currently classified into eight genetic assemblages (A–H) of which assemblages A and B are zoonotic [6]. For *E. Bieneusi*, > 500 distinct genotypes have been reported, and phylogenetic analysis has divided these into 11 distinct groups (groups 1–11), with > 90% of the genotypes belonging to groups 1 or 2 [7]. Some genotypes are found in a variety of animals, including humans, thus indicating their zoonotic potential.

The prevalence and genotype distribution of these three pathogens in sheep and goats has been widely reported [8–10], but most previous studies have involved an intensive farming environment. Tibetan sheep, which are highly adapted to the high altitudes of Qinghai Province and economically important to local herders, are generally raised using a combination of supplementary feeding and semi-stocking. During the growing season (June–October), when natural pasture can provide enough herbage, Tibetan sheep are always raised in free pastures [11]. Recent studies on *Cryptosporidium* spp., *G. duodenalis* and *E. bieneusi* infections in Tibetan sheep have been reported conflicting results [12–15]. To date, there has been no systematic study on the seasonal distribution of these pathogens in Tibetan sheep.

The aim of the present study was to examine the prevalence, genotype characterization and seasonal distribution of *Cryptosporidium* spp., *G. duodenalis* and *E. bieneusi* in Tibetan sheep in Qinghai, China, and to assess the zoonotic transmission potential of these pathogens and their impact on public health.

## Methods

### Sample collection

From May 2016 to August 2017, 761 fecal samples were collected from Tibetan sheep in seven counties in Qinghai Province, China. All samples were collected from grazing sheep with no adverse clinical symptoms. The age difference between sheep was relatively small. As the sheep were raised in a natural pasture, we collected the top layers of the fecal material immediately after defecation, thus avoiding the part in contact with the ground. The sheep were numbered before sampling, and only one fecal sample was collected per animal. These samples were transported to the laboratory under cool conditions and preserved in 2.5% potassium dichromate at 4 °C until DNA extraction.

### DNA extraction

Each fecal sample (0.5 mg) was washed 3 times with distilled water to remove the potassium dichromate. DNA was extracted using the Stool DNA Kit (OMEGA, China) according to the manufacturer’s instructions and then stored at −20 °C until PCR amplification.

### Detection, genotyping and subtyping of *Cryptosporidium* spp.

*Cryptosporidium* spp. were examined by PCR analysis of an approximately 830-bp fragment of the small subunit ribosomal RNA gene (*18S* rRNA) [16]. The *Cryptosporidium* spp. present in the samples were identified to the species level by sequence analysis of the secondary PCR products. *Cryptosporidium ubiquitum* was then subtyped using a PCR assay and sequence analysis of an approximately 850-bp fragment of the 60-kDa glycoprotein gen (*gp60*), as described previously [17].

### Detection, genotyping and subtyping of *G. duodenalis*

Genotyping of *G. duodenalis* was performed by PCR analysis of an approximately 532-bp fragment of triosephosphate isomerase genetic locus (*TPI*) [18]. Assemblages of *G. duodenalis* were determined using sequence analysis of the secondary PCR products.

### Detection, genotyping and subtyping of *E. bieneusi*

*Enterocytozoon bieneusi* was detected by PCR analysis of an approximately 390-bp fragment of the internal transcribed spacer gene (*ITS*) [19]. Genotypes of *E. bieneusi* were determined by sequence analysis of the PCR products.

### DNA sequence analysis

All DNA samples which tested for the pathogens were sent to Sangon Biotech Co., Ltd. (Shanghai (China) for bidirectional DNA sequence analysis. Raw sequences were assembled using DNAStar 5.0 [20] and aligned using Clustal X 1.83 [21], following which the sequences were used to construct a phylogenetic analysis tree using the maximum likelihood (ML) method, with MEGA 7.0.26 software [22]. The Hasegawa-Kishino-Yano (HKY) model and gamma distribution were used to calculate the substitution rates to identify the genotypes of *E. bieneusi*. The reliability of each phylogenetic tree was assessed using a bootstrap analysis with 1000 replicates.

### Statistical analysis

The Chi-square test (χ2 test) was used to determine the relationships between positivity rates and locations of *Cryptosporidium* spp., *G. duodenalis* and *E. bieneusi*, as well as the relationships between the positivity rates and seasons. Statistical analysis was implemented in SPSS software version 20.0 (SPSS IBM, Armonk, NY, USA) for Windows. Differences were considered significant at the 0.05 level.

## Results

### Mixed infection of *Cryptosporidium* spp., *G. duodenalis* and *E. bieneusi* in Tibetan sheep

Five fecal samples were identified having mixed infections. One was positive for both *Cryptosporidium* spp. and *G. duodenalis*; one was positive for both *Cryptosporidium* spp. and *E. bieneusi*; and the remaining three fecal samples contained a mixture of *G. duodenalis* and *E. bieneusi*. However, in none of the samples were all three pathogens detected concurrently.

### Prevalence and seasonal distribution of *Cryptosporidium* spp. in Tibetan sheep

PCR analysis confirmed that 28 (3.68%) of fecal samples collected from Tibetan sheep were positive for *Cryptosporidium* spp. Across the seven counties in Qinghai Province where samples were collected from sheep, *Cryptosporidium* spp. was only found in four counties, where the positivity rates ranged from 2.80% (Huangnan County, 3/107) to 6.13% (Haibei County, 13/212) (Table 1); however, the differences were not statistically significant (*χ*^2^ = 10.18, *df* = 6, *P* ˃ 0.05).

**Table 1:** Prevalence and species/assemblage/genotype distribution of *Cryptosporidium* spp., *Giardia duodenalis* and *Enterocytozoon bieneusi* in Tibetan sheep in Qinghai Province

Variable	No. of samples	*Cryptosporidium* spp.		*Giardia duodenalis*		*Enterocytozoon bieneusi*	
		No. of positive samples (%)	Species (*n*)	No. of positive samples (%)	Assemblages (*n*)	No. of positive samples (%)	Genotype (*n*)
*Location*							
Xining	164	6 (3.66)	*C. xiaoi* (4) *C. ubiquitum* (1) *C. bovis* (1)	5 (3.05)	E (5)	22 (13.41)	BEB6 (9), COS-I (3), CGS1 (1), CHS3 (8), PIGEBITS5 (1)
Haibei	212	13 (6.13)	*C. xiaoi* (4) *C. ubiquitum* (4) *C. bovis* (4) *C. ryanae* (1)	5(2.36)	E (3), A (2)	9 (4.25)	BEB6 (4), COS-I (2), CGS1 (1), CHS3 (2)
Golog	51	-	-	-	-	-	
Hainan	124	6 (4.84)	*C. xiaoi* (5) *C. ubiquitum* (1)	2 (1.61)	E (2)	9 (7.26)	BEB6 (6), COS-I (2), CHS3 (1)
Huangnan	107	3 (2.80)	*C. ubiquitum* (2) *C. bovis* (1)	-	-	4 (3.74)	BEB6 (1), COS-I (3)
Haixi	51	-	-	-	-	4 (7.84)	COS-I (4)
Yushu	52	-	-	-	-	1 (1.92)	BEB6 (1)
* Season*							
Spring	183	6 (3.28)	*C. xiaoi* (6)	9 (4.92)	E (9)	12 (6.56)	BEB6 (4), COS-I (3), CHS3 (5)
Summer	212	16 (7.56)	*C. xiaoi* (4) *C. ubiquitum* (6) *C. bovis* (5) *C. ryanae* (1)	1 (0.47)	E (1)	28 (13.21)	BEB6 (14), COS-I (5), CGS1 (2), CHS3 (6), PIGEBITS5 (1)
Autumn	262	4 (1.53)	*C. xiaoi* (1) *C. ubiquitum* (2) *C. bovis* (1)	2 (0.76)	A (2)	8 (3.05)	BEB6 (3), COS-I (5)
Winter	104	2 (1.92)	*C. xiaoi* (2)	-	-	1 (0.96)	COS-I (1)
* Total*	761	28 (3.68)	*C. xiaoi*(13) *C. ubiquitum* (8) *C. bovis* (6) *C. ryanae* (1)	12 (1.58)	E (10), A (2)	49 (6.44)	BEB6 (21), COS-I (14), CGS1 (2), CHS3 (11), PIGEBITS5 (1)

Samples positive for *Cryptosporidium* spp. were found in all seasons, with the highest rate, 7.56% (16/212), in the summer (Table 1). Across different seasons, the positivity rate of *Cryptosporidium* spp. showed significant differences (*χ*^2^ = 13.36, *df* = 3, *P* < 0.01).

The results of the DNA sequence analysis of the* 18S* rRNA gene products showed that the sequences were highly similar (> 99%) to those of known *Cryptosporidium* spp. Subsequent phylogenetic analysis of these sequences identified four species among the 28 isolates of *Cryptosporidium* spp.: *C. xiaoi* (*n* = 13 samples), *C. ubiquitum* (*n* = 8), *C. bovis* (*n* = 6) and *C. ryanae* (*n* = 1), with *C. xiaoi* being the predominant species (13/28, 46.43%) in Tibetan sheep in Qinghai Province. For *C. ubiquitum*, only three of the eight positive samples were successfully subtyped, yielding subtype XIIa. *Cryptosporidium ryanae* was only detected in one sample, and the sequence showed 100% homology to subtype KT922234 derived from a calf in Ethiopia.

### Prevalence and seasonal distribution of *G. duodenalis* species in Tibetan sheep

Of the 761 fecal samples collected from Tibetan sheep in Qinghai Province, 12 (1.58%) tested positive for *G. duodenalis*. These positive samples came from three counties: Xining (5/164, 3.05%), Haibei (5/212, 2.36%) and Hainan (2/124, 1.61%) (Table 1); however, the differences in positivity rate were not statistically significant (*χ*^2^ = 7.31, *df* = 6, *P* < 0.01).

Positive specimens of *G. duodenalis* were found in three seasons, but not in winter. The positivity rate was higher in spring (4.92%, 9/183) than in summer and autumn, and the differences were statistically significant (*χ*^2^ = 12.60, *df* = 3, *P* < 0.01).

DNA sequence analysis led to the identification of two genotypes, and comparison of the similarity with those from from GenBank data (Additional file 1: Dataset 1) showed > 99% similarity. Two samples showed a similarity of 99.81% to zoonotic assemblage A, and the remaining ten sequences were identical to assemblage E, with similarity to GenBank sequences ranging from 99.43% to 100% after BLAST (Basic Local Alignment Search Tool) analysis.

### Prevalence and seasonal distribution of *E. bieneusi* genotypes in Tibetan sheep

The PCR results on the ITS locus showed that 49 (6.44%) samples from Tibetan sheep were positive for *E. bieneusi*. *Enterocytozoon bieneusi* was detected in samples from all counties except Golog, with positivity rates ranging from 1.92% to 13.41%. The highest positivity rate was detected in Xining (22/164, 13.41%) (Table 1). Analysis showed that the differences in positivity rate were statistically significant (*χ*^2^ = 19.39, *df* = 6, *P* < 0.01).

Positive samples of *E. bieneusi* were found across all seasons, with the highest rate in summer (13.21%, 28/212) (Table 1). The results also showed that the differences in positivity rates of *E. bieneusi* in different seasons were significant (*χ*^2^ = 24.25, *df* = 3, *P* < 0.01).

Comparison of the sequences with those in the GenBank database using BLAST analysis revealed five genotypes: BEB6 (*n* = 21 samples), COS-I (*n* = 14), CHS3 (*n* = 11), CGS1 (*n* = 2) and PIGEBITS5 (*n* = 1). Phylogeny analysis indicated that, with the exception of genotype PIGEBITS5, which belongs to group 1, the remaining genotypes all belonged to group 2.

## Discussion

**Fig. 1:**
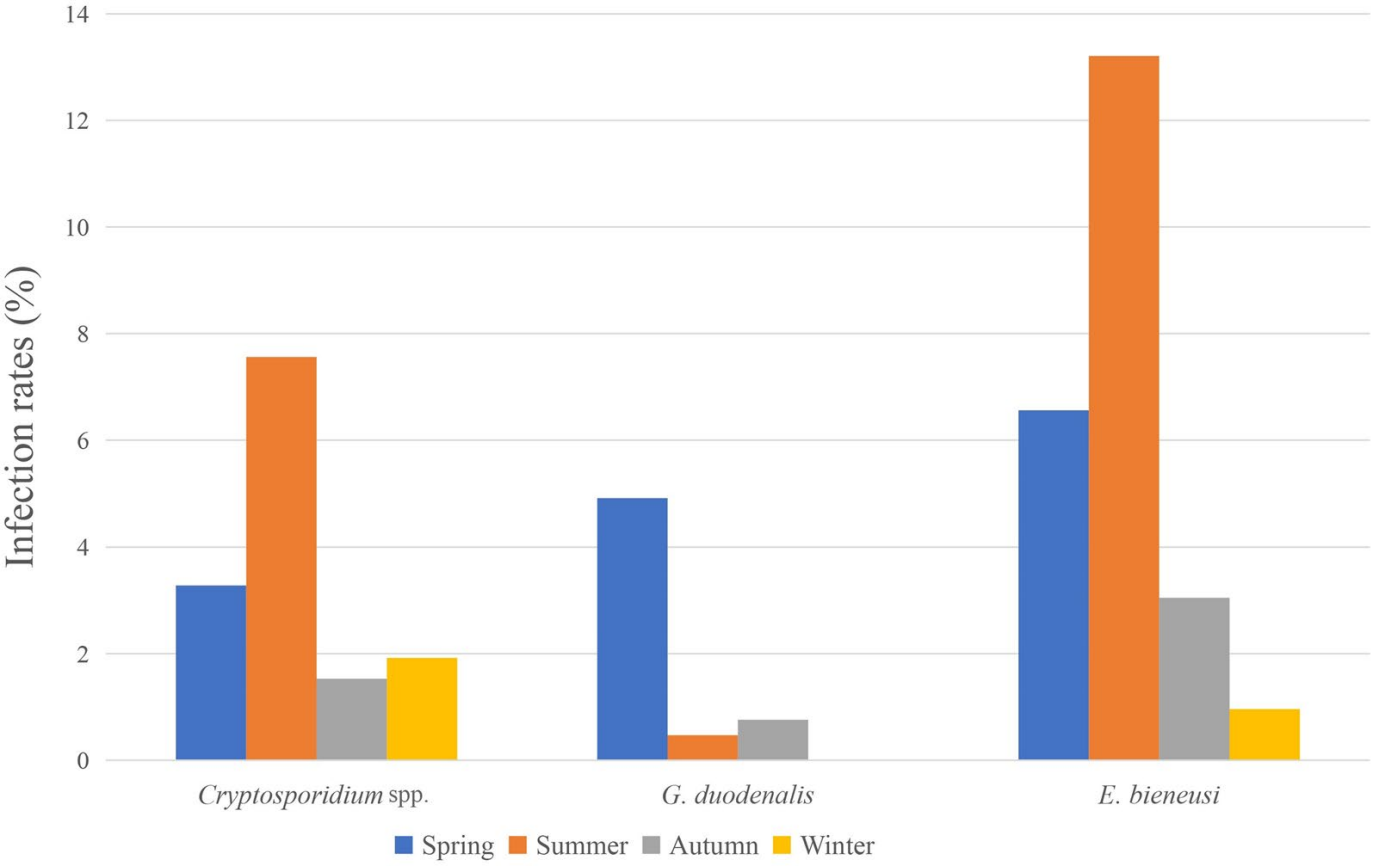
Prevalence of *Cryptosporidium* spp., *Giardia duodenalis* and *Enterocytozoon bieneusi* in Tibetan sheep across the different seasons

In this study, we found that the prevalence of *Cryptosporidium* spp., *G. duodenalis* and *E. bieneusi* in Tibetan sheep was 3.68, 1.58 and 6.44%, respectively. The results of this study showed that the prevalence of these pathogens differed significantly across seasons (Fig. 1). Prior to this study, prevalence data on the seasonal distribution of these pathogens were limited for sheep in China, with the few previous studies reporting on the prevalence of these pathogens in livestock in Ireland, India and Jordan [23–25]. Other related studies mainly focused on humans. The reasons for the seasonal differences observed in the present study are unclear. Many factors, including levels of sunlight and germicidal ultraviolet radiation, environmental temperatures, humidity, breeding density and precipitation, can contribute to such results [26–28].

*Cryptosporidium* spp. are important protozoan parasites that target the gastrointestinal tract of various hosts, including humans, domestic animals and wildlife [29]. In the present study, the overall infection rate of *Cryptosporidium* spp. in Tibetan sheep was 3.68%. In comparison, previous studies reported that the infection rates of *Cryptosporidium* spp. in sheep and goats were between 2.75% and 45.5% in different provinces and cites in China [12, 14, 30–33]. The *Cryptosporidium* spp. infection rate found in the present study is higher than that reported in Papua New Guinea (2.2%) [34] and Egypt (2.5%) [35], but lower than that reported in other countries, such as Greece, Spain, Algeria, Tunisia, Jordan, Poland, Norway and Mexico, where studies reported a wide range, from 5.1% to 67.5% [26, 36–42]. The differences in infection rates between these studies can be attributed to a variety of reasons, such as sample sizes, climate, animal age and animal management methods.

To date, > 10 species of *Cryptosporidium* have been identified in sheep, including *C. xiaoi*, *C. ubiquitum*, *C. parvum*, *C. andersoni*, *C. fayeri*, *C. ryanae, C. scrofarum*, *C. hominis*, *C. suis* and *C. bovis* [30]. In the present study, four *Cryptosporidium* spp. were isolated from Tibetan sheep in Qinghai: *C. xiaoi* (46.43%, 13/28), *C. ubiquitum* (28.57%, 8/28), *C. bovis* (21.43%, 6/28) and *C. ryanae* (3.57%, 1/28). *Cryptosporidium xiaoi* was the dominant species, consistent with previous reports on Tibetan sheep in Qinghai and Inner Mongolia in China [12, 33]. For *C. ubiquitum*, only three isolates were successfully subtyped, among which subtype XIIa has been found in humans and ruminants worldwide. This subtype has also been detected in Tibetan sheep, reflecting its zoonotic potential [43, 44]. Previous studies reported the *C. ryanae* was common in bovines, barking deer, *Cervus uincolor*, buffalo and deer [45]. Our study is the first to detect this species in Tibetan sheep. Mirhashemi et al. detected *C. ryanae* in sheep in Ireland and reported that it was the dominant *Cryptosporidium* species in cattle [25]; it has also been reported in yaks in Qinghai [46]. During summer, which is the growing season, yaks generally share the same pasture with Tibetan sheep; therefore, *C. ryanae* has the potential to spread between yaks and Tibetan sheep, and the animals can infect each other by contaminating the pasture.

Similar to the *Cryptosporidium* spp. infection rates, the infection rates of *G. duodenalis* reported in the present study are drastically different from those reported in previous studies. We found an infection rate in Tibetan sheep of 1.58% which, when compared with rates previously reported in China, are similar to those documented for Tibetan sheep in Gansu (1.7%) [14] and Qinghai (1.3%) [47], but higher than those obtained for Tibetan sheep (0.6%) and goats (0%) in Tibet [48] and sheep in Qinghai (0%) [49]. However, the infection rate is lower than those reported in previous studies on sheep in Heilongjiang (4.3%) [50] and Inner Mongolia (4.3%) [51], and especially the Tibetan sheep in Qinghai (13.1%) [13]. Globally, many researchers have conducted extensive investigations on sheep and goats infected with *G. duodenalis*, and the reported infection rates vary from 1.5% to 55.6% [52, 53]. In addition, in our study there was no significant difference between *G. duodenalis* infections at different altitudes (the altitude variation among the seven sampling counties was 1980 m), which is consistent with the results of a study in the Qinghai-Tibetan Plateau Area (QTPA) (which includes Qinghai, Yunnan and Tibet) [47].

Three assemblages (A, B, E) have been isolated from sheep to date. Assemblage E is the predominant genotype and has a significantly higher prevalence than assemblages A and B [8, 50, 54, 55]. In the present study, sequence comparison showed that two assemblages, E and A, were present in Tibetan sheep. In the past, livestock-specific assemblage E was not considered to be zoonotic as it was mostly detected in sheep, goats, pigs, among others [52]. However, there are emerging reports about this assemblage being detected in three human fecal samples in Egypt [56], and it was subsequently found in persons living in rural settings in Egypt [57], Rio de Janeiro, Brazil [58] and Queensland, Australia [59] and in primates (red colobus) of western Uganda [60]; these results show that assemblage E has zoonotic potential. Therefore, Tibetan sheep herders should be alert to this risk of infection.

In the present study, the infection rate of *E. bieneusi* in Tibetan sheep was 6.44%. Worldwide, several studies have been conducted to identify and assess the prevalence of *E. bieneusi* in sheep and goats. These data are mostly from China [8, 9, 14, 15, 51, 61–63], with a few other reports from Iran [64], Brazil [61] and Sweden [65]. The prevalence of *E. bieneusi* infection in sheep reported in these studies ranges from 4.4% to 69.3%, whereas in goats, it ranges from 7.5% to 32.9%. Three studies reported the infection rates of *E. bieneusi* in Tibetan sheep from Qinghai, Gansu and Tibet in China to be 23.4, 34.5 and 15%, respectively [14, 15, 48]. Compared with the results of the majority of these earlier studies, in our study the infection rate of *E. bieneusi* in Tibetan sheep in Qinghai was relatively lower.

**Fig. 2:**
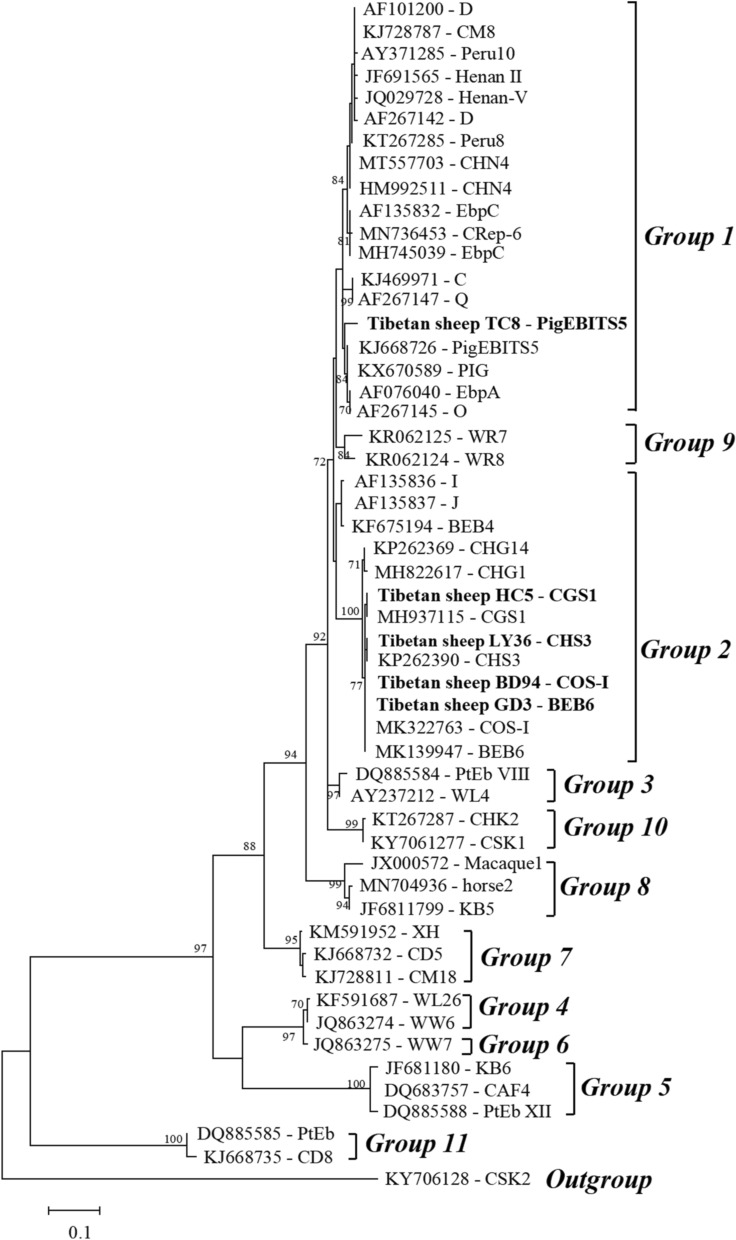
Phylogenetic tree of *Enterocytozoon bieneusi* internal transcribed spacer sequences based on the maximum likelihood method

Many genotypes of *E. bieneusi* have been found in ovines globally through phylogenetic analysis [66]. Most cluster with host-specific groups 1 and 2, which are zoonotic; only the CM4 and CHG21 genotypes belong to group 9 (Table 2). However, many new genotypes are isolated from ovines every year, constantly supplementing the genotype distribution in these animals. In the present study, five genotypes were identified from 49 *E. bieneusi*-positive samples using phylogenetic analysis: BEB6, COS-I, CGS1 and CHS3 belonging to group 2, and PIGEBITS5 belonging to group 1 (Fig. 2). BEB6 (42.9%, 21/49) was the dominant genotype in Tibetan sheep in the present study, which is consistent with the results of previous studies in Qinghai, Henan and Inner Mongolia [9, 15, 51]. CGS1 is a novel genotype that was first identified in Tibetan sheep in Gansu [14]; to date, it has not been isolated from other animals. This new genotype may be a result of host–parasite interactions. Recently, the PIGEBITS5 genotype was found in three Tibetan sheep fecal samples in Tibet [48]. Worldwide, the PIGEBITS5 genotype was first identified in swine in the USA [19]. A subsequent study by Abe and Kimata on pigs in Japan provided strong evidence that the PigEBITS5 genotypes are pig-specific [67], a finding which has been confirmed by many subsequent studies [67–69]. However, this genotype has also been detected in dairy calves [70, 71], *Macaca nemestrina* [72], dogs in China [73] and humans in Czech Republic [74], implying that it may infect a wide range of hosts and is of zoonotic potential.

**Table 2:** Distribution of *Enterocytozoon bieneusi* genotypes in ovines in previous studies

Group	Internal transcribed spacer genotypes
1	BEB19, EbpA, COS-IV, COS-V, COS-VI, COS-VII, D, Peru6, CHG25, CHS5, CHS10, CHS11, CHS12, EbpC, CHG6, CHG7, CHG9, CHG19, CHG23, NESH1, NESH2, NESH3, O, KIN-1, F, COG-I, CHS17, CS-4, LW1, PIGEBITS5
2	BEB6, BEB7, BEB18, CD6, CHG1, CHG2, CHG3, CHG5, CHG8, CHG10, CHG11, CHG12, CHG13, CHG14, CHG16, CHG17, CHG18, CHG20, CHG22, CHG24, CHG25, CHS3, CHS4, CHS6, CHS7, CHS8, CHS9, CHS13, CHS14, CHS15, CHS16, CM7, COS-I, COS-II, COS-III, NESH4, NESH5, NESH6, OEB1, OEB2, SX1, J, I, CGS1
9	CM4, CHG21

## Conclusions

The findings if the present study demonstrate the prevalence, genotype characterization and seasonal distribution of *Cryptosporidium* spp., *G. duodenalis* and *E. bieneusi* in Tibetan sheep in Qinghai Province, China. Four species of *Cryptosporidium* spp. were detected, with *C. xiaoi* being the dominant species, and *Cryptosporidium ryanae* in Tibetan sheep is reported for the first time. The frequency of *G. duodenalis* assemblages E and A showed that the risk of this pathogen to public health in this region may not be high. Furthermore, based on the ITS region, five genotypes of *E. bieneusi* were detected, which clustered into zoonotic phylogenetic groups 1 and 2. This result indicates that Tibetan sheep may be a potential source of zoonotic *E. bieneusi* infection. Systematic analysis was used to detect the seasonal differences for these three protozoan pathogens. More detailed studies are required to assess the zoonotic transmission ability of *Cryptosporidium* spp., *G. duodenalis* and *E. bieneusi* from sheep, and the impact of these pathogens on public health.

## Supplementary Information

**Additional file 1. **Dataset S1. The nucleotide sequences detected on the triosephosphate isomerase (TPI) genetic locus of *Giardia duodenalis*.

## Data Availability

Datasets supporting the conclusions of this article are included within the article. The nucleotide sequences generated in this study were submitted to the GenBank database under the accession numbers OL376571-OL376598 and OL411889-OL411937.

## Abbreviations

BLAST: Basic local alignment search tool
HKY: Hasegawa-Kishino-Yano
ITS: Internal transcribed spacer
ML: Maximum likelihood method
TPI: Triosephosphate isomerase
